# Understanding Side Effects of Antidepressants: Large-scale Longitudinal Study on Social Media Data

**DOI:** 10.2196/26589

**Published:** 2021-03-19

**Authors:** Koustuv Saha, John Torous, Emre Kiciman, Munmun De Choudhury

**Affiliations:** 1 Georgia Institute of Technology Atlanta, GA United States; 2 Department of Psychiatry Beth Israel Deaconess Medical Center Harvard Medical School Boston, MA United States; 3 Microsoft Research Redmond, WA United States

**Keywords:** antidepressants, symptoms, side effects, digital pharmacovigilance, social media, mental health, linguistic markers, digital health

## Abstract

**Background:**

Antidepressants are known to show heterogeneous effects across individuals and conditions, posing challenges to understanding their efficacy in mental health treatment. Social media platforms enable individuals to share their day-to-day concerns with others and thereby can function as unobtrusive, large-scale, and naturalistic data sources to study the longitudinal behavior of individuals taking antidepressants.

**Objective:**

We aim to understand the side effects of antidepressants from naturalistic expressions of individuals on social media.

**Methods:**

On a large-scale Twitter data set of individuals who self-reported using antidepressants, a quasi-experimental study using unsupervised language analysis was conducted to extract keywords that distinguish individuals who improved and who did not improve following the use of antidepressants. The net data set consists of over 8 million Twitter posts made by over 300,000 users in a 4-year period between January 1, 2014, and February 15, 2018.

**Results:**

Five major side effects of antidepressants were studied: sleep, weight, eating, pain, and sexual issues. Social media language revealed keywords related to these side effects. In particular, antidepressants were found to show a spectrum of effects from decrease to increase in each of these side effects.

**Conclusions:**

This work enhances the understanding of the side effects of antidepressants by identifying distinct linguistic markers in the longitudinal social media data of individuals showing the most and least improvement following the self-reported intake of antidepressants. One implication of this work concerns the potential of social media data as an effective means to support digital pharmacovigilance and digital therapeutics. These results can inform clinicians in tailoring their discussion and assessment of side effects and inform patients about what to potentially expect and what may or may not be within the realm of normal aftereffects of antidepressants.

## Introduction

As mental health concerns continue to surge as an epidemic, arguably exacerbated in the present and the near future due to the ongoing COVID-19 pandemic [1], there is a growing need to better understand the impact of antidepressants on individuals with mental illnesses. Typically, the effects of these drugs are measured using randomized controlled trials and databases maintaining adverse event reports [2,3]. However, these trials are susceptible to biases [4]. Importantly, antidepressants are known to show varying effects across individuals and conditions [5]. Despite several meta-analyses [6], existing evaluations of the benefits and harms of antidepressants are based on group data and can only serve as advisories for individual patients. Due to clinical heterogeneity, an individual’s subjective experience is crucially important to consider but is difficult to be incorporated. Understanding the effects of a particular antidepressant on a particular individual is nontrivial, as emphasized in precision psychiatry and Research Domain Criteria–informed treatment research [7]. Common side effects related to antidepressants may include those related to gastrointestinal problems, weight gain or appetite, dry mouth, sleep, and sexual issues among others [8]. Side effects remain a common reason people discontinue these medications [9], yet it remains difficult to anticipate in which patients they will occur. Larger sample sizes are often necessary to uncover new findings about antidepressants [10], and social media offers a new tool to understand side effects that may otherwise remain undetected.

This short paper targets the above gap by adopting an observational study approach using natural language and machine learning methodologies on large-scale social media data. Our work draws motivation from the success of using social media as an effective source of unobtrusive, real-time, low-cost, and naturalistic data to infer wellbeing. Social media platforms enable individuals to share and express their day-to-day psychosocial concerns; therefore, this is a form of longitudinal verbal and behavioral data, and computational linguistic approaches have helped reveal naturalistic patterns of mood, behavior, cognition, psychological state, and social milieu of individuals and collectives [11-15].

Linguistic cues and social interactions on social media have enabled the inference of psychopathologies including depression, anxiety, stress, suicidal ideation, and loneliness [11,12,16-19]. In particular, the public-facing and microblogging-based design of Twitter (which is also the primary data set of our study) is known to enable candid self-disclosure and self-expressions of individuals, including on sensitive topics such as mental health and behavioral symptoms [20,21]. Twitter data were also recently leveraged to measure the psychosocial effects of COVID-19 [20]. Recent data and meta-reviews suggest that people are often more honest and may self-disclose more about mental health concerns and medications on social media than on other mediums [22].

This work uses natural language and causal inference analytic techniques on self-initiated social media expressions of antidepressant users to study the heterogeneity of individual and drug-specific outcomes. Our rationale is situated in the emergent body of empirical evidence in “digital pharmacovigilance” [23]. Digital pharmacovigilance has enabled understanding effects (and side effects) of prescription drugs by employing data mining techniques on large-scale social media data [24]. In recent studies leveraging publicly available Twitter data, Nikfarjam et al studied the mentions of side effects of 81 drugs [25], and Saha et al conducted a quasi-experimental study to explore the effects of 49 generic psychiatric medications [26]. These works revealed that Twitter data are an effective source to study digital pharmacovigilance, including that of antidepressants. This work extends the existing body of work by examining a set of clinically grounded side effects of antidepressants through identifying distinct linguistic markers in the longitudinal Twitter data; these data comprise 112,025,496 posts from 34,518 individuals showing the most and the least improvement in symptoms following the self-reported intake of antidepressants.

## Methods

### Data

To begin with, a list of Food and Drug Administration–approved antidepressants and antidepressant augmentation drugs was developed in consultation with the psychiatrist coauthor (JT). This list included 297 brand names mapped to 49 generic names across the four major drug families: serotonin-norepinephrine reuptake inhibitors (SNRIs), selective serotonin reuptake inhibitors (SSRIs), tricyclic antidepressants, and tetracylic antidepressants. Next, the Twitter application programming interface was queried for public English posts containing the brand name or generic name of these drugs between January 1, 2015, and December 21, 2016, to obtain 601,134 posts by 230,573 unique users. Because a mention of a drug does not necessarily indicate a self-intake, a personal medication intake classifier built in prior work [27] was used to identify self-intakes of these medications. This classifier used a support vector machine model and could distinguish if a Twitter post corresponded to a self-report about a personal medication intake with an accuracy and F1 score of 0.82 [26]. The personal medication intake classifier identified 93,275 posts from our initial data set to indicate a personal intake. After pruning the data to only typical Twitter users, the entire longitudinal Twitter data sets of 23,191 users were collected, amounting to 112,052,496 Twitter posts made in the 4-year period between January 1, 2014, and February 15, 2018. These users self-disclosed the personal intake of 297 brand names (mapping to 49 generic names) of psychiatric medications (Textbox 1). A control data set was also built including 707,475,862 Twitter posts from the same period in the longitudinal timelines of 283,374 random users who did not disclose any medicine intake (also referred to as the control users).

**List of antidepressants considered in this work.**

agomelatine, amineptine, amitriptyline, amoxapine, bupropion, butriptyline, citalopram, clomipramine, desipramine, desvenlafaxine, dibenzepin, dosulepin, doxepin, duloxetine, escitalopram, etoperidone, fluoxetine, fluvoxamine, hydroxynefazodone, imipramine, iprindole, levomilnacipran, lofepramine, maprotiline, mazindol, meta-chlorophenylpiperazine, mianserin, mirtazapine, nefazodone, nisoxetine, nomifensine, norclomipramine, northiaden, nortriptyline, opipramol, oxaprotiline, paroxetine, protriptyline, reboxetine, sertraline, setiptiline, trazodone, triazoledione, trimipramine, venlafaxine, vilazodone, viloxazine, vortioxetine, zimelidine

### Study Design

This work aimed to obtain individuals who most and least improved in mental health symptomatic outcomes after the use of antidepressants. Replicating prior work [26], the study was designed by adopting a quasi-experimental approach to measure the relative treatment effect (RTE) of antidepressants of self-disclosed users of these drugs on social media. This approach draws motivation from the potential outcomes framework [28], where counterfactual outcomes are estimated based on the outcomes of similar (matched) individuals. A stratified propensity score analysis was conducted to match treatment and control, conditioned on a set of covariates. These covariates were computed on the pretreatment data of each user and included social media structural features (number of followers and Twitter posts, duration on platform) and linguistic features such as psycholinguistic use [2], 2000 raw unigrams, and baseline mental health symptomatic outcomes [26]. A logistic regression model predicting a user’s treatment status based on the covariates estimated the propensity scores, and then the propensity scores were stratified into 100 strata of equal length. Thereby, each stratum contained matched treatment and control users who exhibited similar propensity scores. Then, the RTE was quantified for each drug in each strata of matched individuals as the ratio of the likelihood of an outcome measure in the treatment group to that in the control group. The outcome measures consisted of mental health symptomatic expressions of depression, anxiety, stress, suicidal ideation, and psychosis, as quantified via binary transfer learning classifiers of these expressions [26]. Accordingly, for each drug, after sorting the strata in descending order of RTEs, the top 10 strata contained users with the most improvement, and the bottom 10 strata contained users with the least improvement.

To drill into the posttreatment linguistic markers associated with the symptomatic changes per medication, an unsupervised language modeling approach, the Sparse Additive Generative (SAGE) model [29], was employed on the posttreatment Twitter posts of the most and least improved users. Given any two data sets, SAGE selects distinctive keywords from each data set by comparing the parameters of two logistically parameterized multinomial models, using a self-tuned regularization parameter to control the trade-off between frequent and rare terms. SAGE identified the salient *n*-grams in the posttreatment data sets of treatment users who showed contrasting changes in symptomatic outcome measures of depression, anxiety, stress, suicidal ideation, and psychosis. A detailed approach is included in the supplementary information in Multimedia Appendix 1 [2,26,28-30]. This paper compares and reports the analysis for 4 major antidepressants and qualitatively dives deep into the linguistic markers.

## Results

### Overview

**Figure 1:**
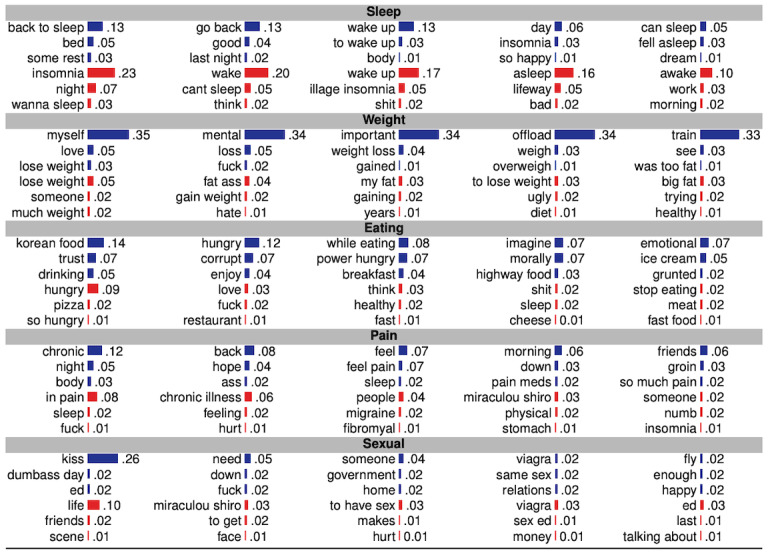
Top n-grams (n=1,2,3) with normalized prevalence that co-occur with the named side effects. Blue and red bars represent magnitude of normalized occurrence in the social media data of individuals showing the most and least improved symptomatic outcomes, respectively.

To understand drug-specific effects, first, the posttreatment linguistic markers associated with the symptomatic outcomes of individuals who used antidepressants were compared (Figure 1). Table 1 shows the linguistic markers from the data sets of those individuals who showed the most improved RTE and those who showed the least improved RTE in outcomes. Table 2 presents the excerpts of five side effects known to be clinically significant for antidepressants, which are explained below.

**Table 1:** Top keywords extracted by SAGE [29] in the most and least improved strata of the 4 most popular antidepressants. Improvement is measured in terms of relative treatment effect on strata of similar users based on changes in mental health symptomatic outcomes of depression, anxiety, stress, suicidal ideation, and psychosis.

Antidepressant	Most improved in symptomatic outcomes	Least improved in symptomatic outcomes
Sertraline	fell asleep, fall asleep, good night, hours sleep, makes sad, asexual, kissing, lips, sex, daddy, honest, fear, love, hair, tonight, makeup, excited, kid, conversation, doctor, wanna talk	fucking kidding, stop listening, conspiracy theory, green tea, math class, times bed, want eat, want sleep, freedom speech, want die, dark souls, dental plan, hell drug, family problems
Escitalopram	weight loss, fall asleep, feeling better, stay safe, want eat, swear god, thank love, making feel, spend time, hours sleep, sleep night, want sleep, lose weight, dreams, want die, best friend	grad school, parents, health care, health insurance, viagra, pre scription, patient, eye, alcohol, 20 mg, weight gain, cancer, weed, sexual, married, anxiety attack, dont want, sex pills
Fluoxetine	eating disorder, mental illness, feel good, mental health, fall asleep, hungry, hair, scared, smell, lose weight, just feel, anxiety, hate people, need help, sleeping, asking friend	work today, really hope, big deal, wanna know, health care, research, girlfriend, husband, work tomorrow, middle class, read book, great day, really bad, trying make, old man
Duloxetine	lives matter, chronic pain, pain meds, weight loss, heart attack, im crying, best friend, hours sleep, mental health, going sleep, sexual, im excited, self care, lose weight, healthy	today cancer, let play, better soon, good night, awake, hope feel better, panic attack, chronic illness, feel sick, pharmacy, weed, brother, hair, smoke, wake, food, bed, tired, beer, married

**Table 2:** Excerpts from individuals who improved the most and least.

Side effect	Excerpt from most improved (antidepressant used)	Excerpt from least improved (antidepressant used)
Sleep	I’m worried I fall asleep literally anywhere. (sertraline)	Badly just want some sleep. It is not happening! (sertraline)
Weight	This drug may cause weight loss and hypomania cool! (duloxetine)	My body is absolutely starting weight gain. It’s terrible (escitalopram)
Eating behavior	I deal with depression, social anxiety, obsessional OCD, and eating disorders, and that’s pretty damn cool (fluoxetine)	i want to eat my body weight in food (duloxetine)
Pain	Come to join others dealing with the daily challenges of chronic illness and chronic pain from the comfort of your life (duloxetine)	I suck at sleeping always, a common chronic pain thing (duloxetine)
Sexual issues	Come at me brother. I could have anyone but I choose to be with who I want so I’m technically asexual until I want intimacy (sertraline)	Even my viagra did not work! (escitalopram)

### Sleep

Antidepressants are known to affect the circadian rhythms [31]. Sleep-related keywords distinctly occurred in the data sets of the most and least improved antidepressant users. For instance, for sertraline, the most improved users distinctly used “fall asleep,” whereas the least improved users used “sleep paralysis” and “want sleep.” Similar contrasting patterns were found in duloxetine, where the most improved users distinctly used “going sleep,” whereas the least improved users used “wake” and “awake.” The contrasting effects are also evident in Table 2.

### Weight

Change in weight is a prominent behavioral side effect of many psychiatric medications [9]. The keywords of “weight loss” and “lose weight” dominated in the data set of the improved users of escitalopram, fluoxetine, and duloxetine, whereas “weight gain” dominated in the least improved users of escitalopram.

### Eating Behavior

Keywords related to eating occurred saliently in both the most and least improved users. For instance, keywords such as “eating disorder” and “hungry” dominated in the posts by the improved users of fluoxetine, whereas “want eat” dominated in the least improved users of sertraline, and “food” dominated in the least improved users of duloxetine. 

### Pain

Neuropathic pain is a symptom in depression [32] and is also often comorbid with many depressive disorders. Additionally, antidepressants such as duloxetine are prescribed for chronic pain–related complications [32,33]. These factors could explain the salience in related keywords such as “chronic pain” and “chronic illness” in response to reactions to duloxetine.

### Sexual Issues

Antidepressants are likely to affect sexual issues [34]. In this regard, our data set reveals varying findings; keywords such as “sex,” “love,” “kissing,” and “asexual” dominate in the most improved users of sertraline, whereas “viagra” and “sexual” dominate in the least improved users of escitalopram.

## Discussion

Summarily, our linguistic analysis reveals many keywords potentially related to prominent side effects of the antidepressants. There is a lack of simple and systematic means to understand side effects of antidepressants [4,5]. It is known that many side effects remain underreported in clinical care, and this study offers a new means to better assess what the actual burden and lived experience of patients may be. A major takeaway of our work is that examining social media data corresponding to self-reported medication use and symptomatic outcomes, can enable the discovery of drug-specific effects and adverse effects, including personal accounts of how these effects can impact the lives of individuals. For instance, mentions of sleep were found to be largely associated with improvements following reported use of certain medications. In the context of weight, self-experiences of gaining weight following medication intake, as well as using antidepressants to target weight loss, were found. These potential effects of antidepressants are known in the literature [35], especially a recent finding related to the last observation, which is that certain antidepressants contain glucose-lowering agents that lead to weight loss [35,36]. However, in some cases, the same keyword appeared in both most and least improved outcomes, such as “sleep” for sertraline and “pain” for duloxetine. This observation also aligns with clinical studies that report many psychiatric medications to have both sleep-disturbing and sleep-promoting effects [37]. 

Our knowledge about the etiopathogenesis of mental diseases continues to be “top to bottom” instead of being “bottom up” [7]. Considering that the efficacy of all antidepressants is roughly the same and that prescribing is often done based on the side effect profiles of these medications [5], there is an urgent need to better understand the common effects of antidepressants. These observations of effects are able to produce observable data reflecting an individual’s perceived effect and, at the same time, provide scalable population level insights. While there remain many concerns in using social media data in clinical care, recognizing value in social media data and how they may help inform decision making is an important step. Consequently, translating the potential promised by this brief research into impacting real world outcomes and decisions needs engaging conversations between computational and clinical researchers and practitioners, as well as policymakers. This work can be used to inform algorithms in the clinical decision support modules of medical records to help ensure that prescribing is more tailored to actual side effects of antidepressants. This study can also help inform patients about the full range of side effects and be used as a tool for more standard decision making around medication selection. In addition, this work bears implications toward drug repurposing and in developing new drugs that target treatments with fewer side effects.

Finally, this study adopts a quasi-experimental study design, which cannot establish a “true causality.” However, it is more robust than more simple correlational analysis because of minimizing the confounders. This work motivates future computational study designs that combine the power of complementary digital data sources, including social media, and other ubiquitous data streams to obtain a more holistic understanding of an individual’s behavior with respect to the use of antidepressants.

## Appendix

Multimedia Appendix 1 Supplementary information.

## Abbreviations

RTE: relative treatment effect
SAGE: Sparse Additive Generative
SNRI: serotonin-norepinephrine reuptake inhibitor
SSRI: selective serotonin reuptake inhibitor

## References

[1] Holmes EA, O'Connor RC, Perry VH, Tracey I, Wessely S, Arseneault L, Ballard C, Christensen H, Cohen Silver R, Everall I, Ford T, John A, Kabir T, King K, Madan I, Michie S, Przybylski AK, Shafran R, Sweeney A, Worthman CM, Yardley L, Cowan K, Cope C, Hotopf M, Bullmore E (2020). Multidisciplinary research priorities for the COVID-19 pandemic: a call for action for mental health science. Lancet Psychiatry.

[2] Tausczik Y, Pennebaker J (2009). The Psychological Meaning of Words: LIWC and Computerized Text Analysis Methods. Journal of Language and Social Psychology.

[3] Cipriani A, Furukawa T, Salanti G, Chaimani A, Atkinson L, Ogawa Y, Leucht S, Ruhe H, Turner E, Higgins J, Egger M, Takeshima N, Hayasaka Y, Imai H, Shinohara K, Tajika A, Ioannidis J, Geddes J (2018). Comparative Efficacy and Acceptability of 21 Antidepressant Drugs for the Acute Treatment of Adults With Major Depressive Disorder: A Systematic Review and Network Meta-Analysis. Focus (Am Psychiatr Publ).

[4] Lexchin J, Bero LA, Djulbegovic B, Clark O (2003). Pharmaceutical industry sponsorship and research outcome and quality: systematic review. BMJ.

[5] Gøtzsche Peter C, Young AH, Crace J (2015). Does long term use of psychiatric drugs cause more harm than good?. BMJ.

[6] Fournier JC, DeRubeis RJ, Hollon SD, Dimidjian S, Amsterdam JD, Shelton RC, Fawcett J (2010). Antidepressant drug effects and depression severity: a patient-level meta-analysis. JAMA.

[7] Insel TR (2014). The NIMH Research Domain Criteria (RDoC) Project: precision medicine for psychiatry. Am J Psychiatry.

[8] Wang S, Han C, Bahk W, Lee S, Patkar AA, Masand PS, Pae C (2018). Addressing the Side Effects of Contemporary Antidepressant Drugs: A Comprehensive Review. Chonnam Med J.

[9] Cartwright C, Gibson K, Read J, Cowan O, Dehar T (2016). Long-term antidepressant use: patient perspectives of benefits and adverse effects. Patient Prefer Adherence.

[10] Anderson KN, Lind JN, Simeone RM, Bobo WV, Mitchell AA, Riehle-Colarusso T, Polen KN, Reefhuis J (2020). Maternal Use of Specific Antidepressant Medications During Early Pregnancy and the Risk of Selected Birth Defects. JAMA Psychiatry.

[11] De Choudhury M, Gamon M, Counts S (2011). Predicting depression via social media. https://ojs.aaai.org/index.php/ICWSM/article/view/14432.

[12] Chancellor S, De Choudhury M (2020). Methods in predictive techniques for mental health status on social media: a critical review. NPJ Digit Med.

[13] Jaidka K, Giorgi S, Schwartz HA, Kern ML, Ungar LH, Eichstaedt JC (2020). Estimating geographic subjective well-being from Twitter: A comparison of dictionary and data-driven language methods. Proc Natl Acad Sci U S A.

[14] Schwartz HA, Eichstaedt JC, Kern ML, Dziurzynski L, Ramones SM, Agrawal M, Shah A, Kosinski M, Stillwell D, Seligman MEP, Ungar LH (2013). Personality, gender, and age in the language of social media: the open-vocabulary approach. PLoS One.

[15] Golder SA, Macy MW (2013). Social media as a research environment. Cyberpsychol Behav Soc Netw.

[16] Merchant RM, Asch DA, Crutchley P, Ungar LH, Guntuku SC, Eichstaedt JC, Hill S, Padrez K, Smith RJ, Schwartz HA (2019). Evaluating the predictability of medical conditions from social media posts. PLoS One.

[17] Guntuku SC, Schneider R, Pelullo A, Young J, Wong V, Ungar L, Polsky D, Volpp KG, Merchant R (2019). Studying expressions of loneliness in individuals using twitter: an observational study. BMJ Open.

[18] Coppersmith G, Leary R, Crutchley P, Fine A (2018). Natural Language Processing of Social Media as Screening for Suicide Risk. Biomed Inform Insights.

[19] Saha K, Chan L, De Barbaro K, Abowd GD, De Choudhury M (2017). Inferring Mood Instability on Social Media by Leveraging Ecological Momentary Assessments. Proc ACM Interact Mob Wearable Ubiquitous Technol.

[20] Saha K, Torous J, Caine ED, De Choudhury M (2020). Psychosocial Effects of the COVID-19 Pandemic: Large-scale Quasi-Experimental Study on Social Media. J Med Internet Res.

[21] Ernala S, Labetoulle T, Bane F, Birnbaum M, Rizvi A, Kane J, De Choudhury M (2018). Characterizing Audience Engagement and Assessing Its Impact on Social Media Disclosures of Mental Illnesses. https://ojs.aaai.org/index.php/ICWSM/article/view/15027.

[22] Seabrook EM, Kern ML, Rickard NS (2016). Social Networking Sites, Depression, and Anxiety: A Systematic Review. JMIR Ment Health.

[23] World Health Organization (2002). The Importance of Pharmacovigilance.

[24] Härmark L, van Grootheest AC (2008). Pharmacovigilance: methods, recent developments and future perspectives. Eur J Clin Pharmacol.

[25] Nikfarjam A, Sarker A, O'Connor K, Ginn R, Gonzalez G (2015). Pharmacovigilance from social media: mining adverse drug reaction mentions using sequence labeling with word embedding cluster features. J Am Med Inform Assoc.

[26] Saha K, Sugar B, Torous J, Abrahao B, Kıcıman E, De Choudhury M (2019). A Social Media Study on the Effects of Psychiatric Medication Use. Proc Int AAAI Conf Weblogs Soc Media.

[27] Klein A, Sarker A, Rouhizadeh M, O'Connor K, Gonzalez G (2017). Detecting personal medication intake in Twitter: an annotated corpus and baseline classification system.

[28] Imbens G, Rubin D (2015). Causal Inference in Statistics, Social, and Biomedical Sciences.

[29] Eisenstein J, Ahmed A, Xing E (2011). Sparse additive generative models of text.

[30] Saha K, De Choudhury M (2017). Modeling Stress with Social Media Around Incidents of Gun Violence on College Campuses. Proc ACM Hum Comput Interact.

[31] Mayers AG, Baldwin DS (2005). Antidepressants and their effect on sleep. Hum Psychopharmacol.

[32] Sansone RA, Sansone LA (2008). Pain, pain, go away: antidepressants and pain management. Psychiatry (Edgmont).

[33] Lunn MPT, Hughes RAC, Wiffen PJ (2014). Duloxetine for treating painful neuropathy, chronic pain or fibromyalgia. Cochrane Database Syst Rev.

[34] Montejo A, Llorca G, Izquierdo J, Rico-Villademoros F (2001). Incidence of sexual dysfunction associated with antidepressant agents: a prospective multicenter study of 1022 outpatients. Spanish Working Group for the Study of Psychotropic-Related Sexual Dysfunction. J Clin Psychiatry.

[35] Fava M (2000). Weight gain and antidepressants. J Clin Psychiatry.

[36] Brown A, Rasooly D, Patel C (2018). Leveraging Population-Based Clinical Quantitative Phenotyping for Drug Repositioning. CPT Pharmacometrics Syst Pharmacol.

[37] Wilson S, Argyropoulos S (2005). Antidepressants and sleep: a qualitative review of the literature. Drugs.

