# Supplementary material for: Understanding Side Effects of Antidepressants: Large-scale Longitudinal Study on Social Media Data
Source: JMIR Ment Health. 2021 Mar 19;8(3):e26589. doi: 10.2196/26589 (PMC8077932; doi:10.2196/26589)
Supplement: Multimedia Appendix 1 [file mental_v8i3e26589_app1.docx]

## Supplementary Information

The study design aimed at measuring the effects of psychiatric medications. Outcomes were defined as mental health symptomatic measures of depression, anxiety, stress, suicidal ideation, and psychosis. For each of these symptomatic outcomes, we used an n-gram based classifier that infers if a social media post indicates an expression of the corresponding symptom. For example, a post such as “I am feeling depressed” on social media would be identified to contain language indicative of depression. These classifiers were built using a transfer learning [1, 2] methodology where the groundtruth data were obtained from Reddit communities of these symptoms (e.g., r/depression for depression, r/anxiety for anxiety, r/stress for stress, etc.). These classifiers performed at a high accuracy of approximately 0.90 on held-out test data [1]. These classifiers were also shown to transfer well with an 87% agreement between machine-predicted labels and expert appraisals, where experts annotated posts using DSM-5 criteria of mental health symptoms. We note that the top features of these classifiers do not coincide with the named side effects examined in this work, therefore, minimizing the likelihood that our observations are biased by the classifiers.

The study design adopted a quasi-experimental framework based on matching, which simulates an RCT setting by minimizing for high-dimensional observed covariates [3]. This approach draws motivation from the potential outcomes framework [3], where counterfactual outcomes are estimated based on the outcomes of similar (matched) individuals. We employed stratified propensity score analysis to match Treatment and Control, conditioned on a set of covariates. These covariates were computed on the pre-treatment data of each user and included social media structural features (number of followers and Twitter posts, duration on platform), and linguistic features such as psycholinguistic use [4], 2,000 raw unigrams, and baseline mental health symptomatic outcomes. A logistic regression model predicting a user’s treatment status based on the covariates estimated the propensity scores, and then the propensity scores were stratified into 100 strata of equal length. Thereby, each strata contained matched Treatment and Control users who exhibited similar propensity scores. The quality of matching was evaluated by computing the standardized mean differences (SMD) across the covariates in the Treatment and Control groups, which showed a significant drop of mean SMD from 0.029 to 0.009 from the unmatched to matched data sets, enabling confidence in the quality of matching.

## References

1. Saha, K., Sugar, B., Torous, J., Abrahao, B., Kıcıman, E. and De Choudhury, M. (2019) A social media study on the effects of psychiatric medication use. *Proceedings of the International AAAI Conference on Web and Social Media*, **13**, 440–451. URL: https://www.aaai.org/ojs/index.php/ICWSM/article/view/3242.

2. Saha, K. and De Choudhury, M. (2017) Modeling stress with social media around incidents of gun violence on college campuses. *Proc. ACM Hum.-Comput. Interact.*, **1**. URL: https://doi.org/10.1145/3134727.

3. Imbens, G. W. and Rubin, D. B. (2015) *Causal inference in statistics, social, and biomedical sciences*. Cambridge.

4. Tausczik, Y. R. and Pennebaker, J.W. (2010) The psychological meaning of words: Liwc and computerized text analysis methods. *Journal of language and social psychology*, **29**, 24–54.

5. Eisenstein, J., Ahmed, A. and Xing, E. P. (2011) Sparse additive generative models of text.
